# Oxidative Stress Markers Are Associated with a Poor Prognosis in Patients with Pancreatic Cancer

**DOI:** 10.3390/antiox11040759

**Published:** 2022-04-11

**Authors:** Miguel A. Ortega, Oscar Fraile-Martinez, Leonel Pekarek, Cielo García-Montero, Miguel Angel Alvarez-Mon, Alejandro J. Castellanos, Natalio García-Honduvilla, Julia Buján, Melchor Alvarez-Mon, Miguel A. Sáez, Luis G. Guijarro, Angel Asúnsolo

**Affiliations:** 1 Department of Medicine and Medical Specialities, Faculty of Medicine and Health Sciences, University of Alcalá, 28801 Alcala de Henares, Spain; oscarfra.7@hotmail.com (O.F.-M.); leonel.pekarek@gmail.com (L.P.); cielo.gmontero@gmail.com (C.G.-M.); maalvarezdemon@icloud.com (M.A.A.-M.); alejandrojcastel@gmail.com (A.J.C.); natalio.garcia@uah.es (N.G.-H.); mjulia.bujan@uah.es (J.B.); mademons@gmail.com (M.A.-M.); angel.asunsolo@uah.es (A.A.); 2 Ramón y Cajal Institute of Sanitary Research (IRYCIS), 28034 Madrid, Spain; luis.gonzalez@uah.es; 3 Cancer Registry and Pathology Department, Principe de Asturias University Hospital, 28806 Alcala de Henares, Spain; 4 Oncology Service, Guadalajara University Hospital, 19002 Guadalajara, Spain; 5 Immune System Diseases-Rheumatology, Oncology Service an Internal Medicine, University Hospital Príncipe de Asturias, 28806 Alcala de Henares, Spain; 6 Biomedical Research Networking Center of Hepatic and Digestive Diseases (CIBEREHD), Institute of Health Carlos III, 28034 Madrid, Spain; 7 Pathological Anatomy Service, Central University Hospital of Defence-UAH Madrid, 28801 Alcala de Henares, Spain; 8 Unit of Biochemistry and Molecular Biology, Department of System Biology, University of Alcalá, 28801 Alcala de Henares, Spain; 9 Department of Surgery, Medical and Social Sciences, Faculty of Medicine and Health Sciences, University of Alcalá, 28801 Alcala de Henares, Spain

**Keywords:** pancreatic cancer, survival, biomarkers, PARP1, NOX1, prognosis

## Abstract

Pancreatic cancer is a malignancy of rising prevalence, especially in developed countries where dietary patterns and sedentariness favor its onset. This malady ranks seventh in cancer-related deaths in the world, although it is expected to rank second in the coming years, behind lung cancer. The low survival rate is due to the asymptomatic course of the early stages, which in many cases leads to metastases when becoming evident in advanced stages. In this context, molecular pathology is on the way towards finding new approaches with biomarkers that allow a better prognosis and monitoring of patients. So the present study aims to evaluate a series of molecular biomarkers, PARP1, NOX1, NOX2, eNOS and iNOS, as promising candidates for prognosis and survival by using immunohistochemistry. The analysis performed in 41 patients with pancreatic cancer showed a correlation between a high expression of all these components with a low survival rate, with high statistical power for all. In addition, a 60-month longitudinal surveillance program was managed, accompanied by several clinical parameters. The derivative Kaplan–Meier curves indicated a low cumulative survival rate as well. Ultimately, our research emphasized the value of these molecules as survival-associated biomarkers in pancreatic cancer, offering new gates for clinical management.

## 1. Introduction

Pancreatic cancer is a fatal malady with a 5-year survival rate in approximately 9% of patients [1,2]. The incidence, registered in the GLOBOCAN 2020 fact sheets, was 495,773 new cases worldwide, and keeps ranking 7th place in the number of cancer-related deaths [3]. In any case, the figures are increasing, and it is expected that by 2030 it will be the second-leading cause of cancer-related death in the world, making it a public health burden [4,5]. Some associated risk factors fit with the lifestyle habits of societies in developed countries: sedentariness, heavy alcohol consumption, smoking, elevated levels of glucose and lipids, processed food intake, metabolic and inflammatory diseases such as obesity and diabetes, and stress [6,7].

The challenges faced by this type of cancer lie in the difficulty of providing an effective preventive method. Currently, it is difficult to make an early diagnosis since this cancer goes unnoticed and seldom manifests symptoms in the early stages, only vague and confusing symptoms such as pain in the abdomen or back, and jaundice [8,9]; and what is more serious, it is highly invasive [10,11]. Moreover, the anatomical location of the pancreas complicates the visualization and staging through ultrasonography [12]. This fact makes it even harder to perform surgery. When applied early, surgery is considered the only potentially curative treatment in a minority with a non-metastatic resectable tumor [13], although there is high risk of post-operative morbidity [14]. The rapidity of spreading explains the poor prognosis and low survival rate. It remains as one of the most lethal malignant neoplasms, becoming evident in advanced stages with metastases in most cases [2,15].

In recent years, the focus has been set on screening approaches with the objective of firstly, preventing lethality and improving prognosis, and secondly and ideally, preventing the onset. The only advancements have established high-risk patients that could benefit from screening programs who have pre-malignant conditions such as pancreatic intraepithelial neoplasia, intraductal papillary mucinous neoplasms and mucinous cystic neoplasms [16]. Therefore, the preference for biomarkers that allow early detection remains in the spotlight. In this research area, histopathological studies have been key for advancing the knowledge before the translational clinical management of these kinds of tumors [17,18,19].

With the aim of shedding light on the optimal use of biomarkers, in the present study, we expose the analysis conducted evaluating oxidative stress markers in pancreatic cancer histopathology, and then their relationship with survival rates through longitudinal surveillance programs with clinical parameters. Several authors have already proposed oxidative stress markers as targets for cancer prognosis and treatment due to their role in producing reactive oxygen species (ROS) which cause cell damage and mutagenic metabolites [20]. We mainly focus on five molecules which are characteristic of oxidative stress: poly-ADP-ribosyltransferase (PARP1), NADPH oxidase 1 (NOX1), cytochrome b-245 beta chain (CYBB, but also known as NOX2), endothelial nitric oxide synthase (eNOS) and inducible nitric oxide synthase (iNOS).

Firstly, PARP1 is responsible for the poly-ADP-ribosylation of certain proteins and mediates DNA repair as well. This protein has already been linked to pancreatic cancer tumorigenesis. Redox imbalance produced by elevated oxidorreductases activity is related to DNA damage and stimulates PARP1 [21]. For its part, the Nox family of enzymes contributes to ROS generation as well and promotes the remodeling of the extracellular matrix. The overproduction of ROS has been closely linked to the survival and growth of cancer cells. Some isoforms such as NOX1, which has an important role in regulating H+ currents, have been found to be related to Ras oncogene-induced cell transformation [22]. Lastly, we also included oxide nitric synthases in our analysis: eNOS as a modulator of vascular smooth muscle relaxation, regulating vascular endothelial growth factor (VEGF) and angiogenesis [23]; and iNOS, which is an inducible isoform and key in inflamed tissue. Both are sources of nitro-oxidative stress and seem to be related to oncogenic transformation from inflamed tissue, participating in angiogenesis, invasion and metastasis [24,25].

In this context, the present study will conduct a histopathological (through immunohistochemical techniques) and statistical analysis with the aim to identify a possible role of oxidative stress components as prognostic biomarkers in pancreatic cancer.

## 2. Patients and Methods

### 2.1. Study Design and Sample Collection

The present work is an observational, analytical, retrospective cohort study with a longitudinal follow-up (60 months). Tissue samples were obtained from 41 patients diagnosed with pancreatic cancer who underwent surgery (curative resection of pancreatoduodenectomy). The paraffin blocks and the different details with extensive clinical information about the patients and the follow-up data were retrospectively reviewed. The diagnosis was conducted following the principles of Esposito et al. [26].

The study was performed in agreement with the basic ethical principles of autonomy, beneficence, non-maleficence and distributive justice, and its development fulfilled the rules of Good Clinical Practice, the principles contained in the most recent Declaration of Helsinki (2013) and the Convention of Oviedo (1997). The data and information collected complied with current legislation on data protection (Organic Law 3/2018 of 5 December, Protection of Personal Data and Guarantee of Digital Rights and Regulation (EU) 2016/679). The study was approved by the Hospital Universitario Principe de Asturias (LIB26/2020)-UAH Madrid.

### 2.2. Histopathological and Immunohistochemical Studies

Immunohistochemical studies were conducted on paraffin-embedded pancreatic tissue samples. The antibody recovery step was described in the protocol specifications (Table 1). Antigen/antibody reactions were detected through the avidin–biotin (ABC) complex method, with avidin–peroxidase, according to established protocols [27]. After incubation with the primary antibody (1 h 30 min), samples were incubated with a 3% BSA blocker (Catalog #37525; Thermo Fisher Scientific, Inc., Waltham, MA, USA) and PBS overnight at 4 °C. The samples were incubated with a biotin-conjugated secondary antibody, and then diluted in PBS for 90 min at room temperature ((rabbit IgG, diluted 1/300 (RG-96; Sigma-Aldrich, St. Louis, MI, USA), goat IgG with 1/100 dilution (GT-34/ B3148; Sigma-Aldrich) and mouse IgG with 1/300 diluted (F2012/045K6072; Sigma-Aldrich)). The avidin–peroxidase conjugate ExtrAvidin^®^-Peroxidase (Sigma-Aldrich; Merck KGaA, Darmstadt, Germany) was used for 60 min at RT (1:200 with PBS). Then, protein expression was determined using a Chromogenic Diaminobenzidine (DAB) Substrate Kit (cat. no. SK-4100; Maravai LifeSciences, San Diego, CA, USA), prepared just before exposure (5 mL of distilled water, 2 drops of buffer, four drops of DAB, and two drops of hydrogen peroxide). The signal was achieved with the chromogenic peroxidase substrate for 15 min at RT, allowing the detection of a brown stain. Sections of the same tissue were assigned as negative controls for the detection of each protein, substituting incubation with primary antibody for a PBS as blocking solution. In every tissue, the contrast was performed with Carazzi hematoxylin for 15 min at RT.

### 2.3. Histopathological Assessment

Tissue sections were visualized using a Zeiss Axiophot light microscope (Carl Zeiss, Oberkochen, Germany) equipped with an AxioCam HRc digital camera (Carl Zeiss, Oberkochen, Germany). The histological evaluation was carried out according to the intensity of the expression of the immunohistochemical staining with the IRS-Score method [28]. Therefore, histological samples from patients diagnosed with pancreatic cancer were classified as negative expression (0), low/moderate (1) and high (3). For every group of subjects, seven randomly selected microscopy fields were examined in each of the five sections. Positive individuals were classified when the mean proportion of the labeled sample was superior or equal to 5% of the total sample. This was achieved by calculating the total percentage of marked tissue in the different microscopy fields to obtain an average of the study sample as described in previous works [29]. The quantification and observation of the different samples were performed separately by two independent researchers.

### 2.4. Statistical Analysis

A normality test of markers was carried out (Kolmogorov–Smirnoff, all *p* < 0.001). As we could observe that they did not follow a normal distribution, the results had to be described with medians and interquartile ranges, performing non-parametric tests. A Mann–Whitney U test was used. Simultaneously, in order to assess the association between clinicopathological and immunohistochemical parameters, a logarithmic rank test and Kaplan–Meier curves were developed for survival comparisons. Finally, to explore the correlation of the studied immunohistochemical parameters and the established prognosis of the variables, a univariate analysis and Cox proportional-hazards regression analysis were used. All statistical analyses were performed using STATA 16.1 software (Normal, IL, USA). *p*-values < 0.05 were considered significant.

## 3. Results

### 3.1. Clinical and Sociodemographic Characteristics of the Studied Population

This is as an observational, longitudinal, analytical and retrospective cohort study in which a total of 41 patients were studied (27 men and 14 women, median age = 72.00 [45.00–88.00] years). 28 patients were at a <IV tumor stage, whereas 13 patients were at the IV stage. Patients displayed a median expression for Ca19.9 of 102.10 [44.91–805.00] U/mL, 5.43 [2.71–11.31] ng/mL for CEA, and it was 2.32 [1.46–4.39] ng/mL for AFP. Overall, the survival of patients diagnosed with pancreatic cancer was 8.00 [2.98–13.02] months.

### 3.2. Patients with High Levels of PARP Show a Greater Mortality to Pancreatic Cancer

**Figure 1:**
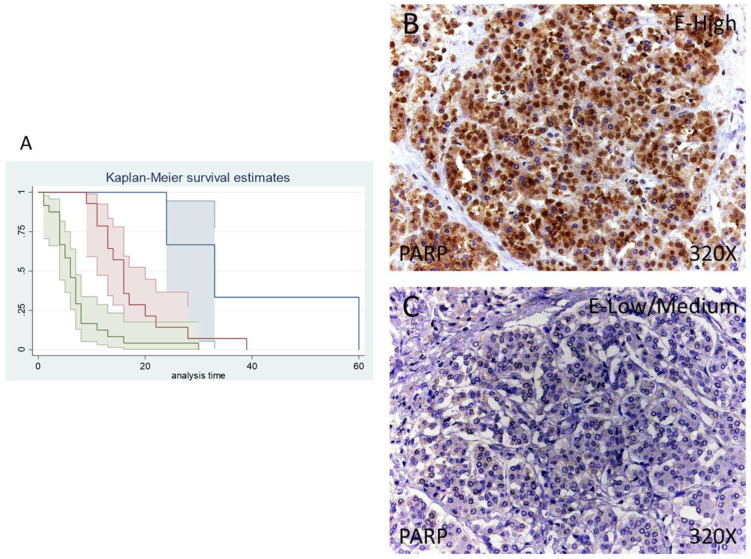
(**A**) Kaplan–Meier curves for survival time according to tumor expression of PARP. Blue curve = tissue expression classified as negative, red curve = tissue expression classified as low/medium, green curve = tissue expression classified as high. (**B**,**C**) Images showing the protein expression of PARP in patients diagnosed with pancreatic cancer.

According to immunohistochemical studies, 92.68% of patients with pancreatic cancer exhibited tissue expression of PARP-1, whereas 7.32% of patients did not show tissue expression of this component. Regarding PARP-1-positive patients, 34.15% of them showed a low/moderate expression whereas 58.53% presented high levels of PARP-1 (Figure 1A,B).

Median survival for patients with pancreatic cancer and a negative tissue expression of IRS 4 was 33 (24–60) months. Patients with a low /moderate expression had a median of 16.00 (11–20) months, whereas in case of patients with a high expression it falls to 6 (4–7) months. According to our statistical associations, having a high expression of PARP-1 is correlated with a hazard ratio of 2.414 of mortality in comparison to a low/moderate expression of this component. The global comparisons showed how the significance value was *p* < 0.001.

### 3.3. Patients with Increased NOX1 and NOX2 Expression Display a Poorer Prognosis

**Figure 2:**
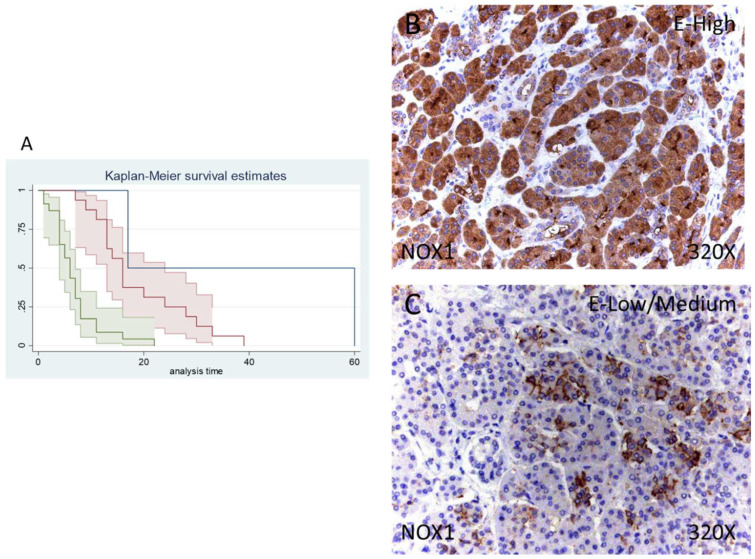
(**A**) Kaplan–Meier curves for survival time according to tumor expression of NOX1. Blue curve = tissue expression classified as negative, red curve = tissue expression classified as low/medium, green curve = tissue expression classified as high. (**B**,**C**) Images showing the protein expression of NOX1 in patients diagnosed with pancreatic cancer.

Of 41 patients, 39 were positive for NOX1 expression (95.12%) whereas 2 (4.88%) were negative for this component. Following our immunohistochemical results, 39.02% of patients exhibited low/moderate and 56.98% high NOX1 expression (Figure 2B,C).

Median survival of patients with a negative expression of NOX1 was 17 (17–60) months, whereas low/moderate NOX1 was 16 (13–24) months and high NOX1 was 6 (4–7) months. There was a hazard ratio of 2.765 of mortality in patients with a high NOX1 expression in comparison to those with a low/moderate expression. The global comparisons defined how the significance value was *p* < 0.001.

**Figure 3:**
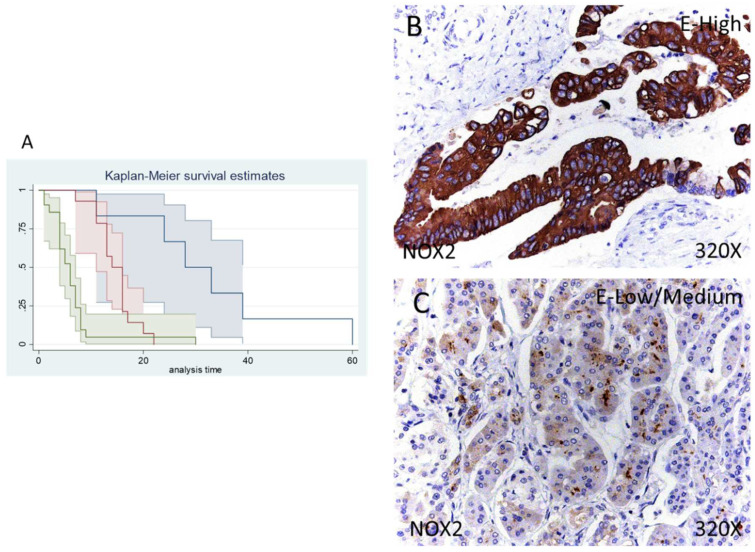
(**A**) Kaplan–Meier curves for survival time according to tumor expression of NOX1. Blue curve = tissue expression classified as negative, red curve = tissue expression classified as low/medium, green curve = tissue expression classified as high. (**B**,**C**) Images showing the protein expression of NOX1 in patients diagnosed with pancreatic cancer.

In the case of NOX2 the percentage of patients with no expression of this component was 14.63%, whereas 34.15% of patients were low/moderate for NOX2 expression and 51.22% displayed a high NOX2 expression (Figure 3B,C).

Median survival of patients with a negative expression of NOX2 was 28 (11–60) months, whereas low/moderate NOX2 was 14 (11–16) months and high NOX2 was 6 (4–7) months. Following our statistical associations, there was a hazard ratio of 2.261 of mortality in patients with a high NOX2 expression in comparison to those with a low/moderate expression. The global comparisons displayed how the significance value was *p* < 0.001

### 3.4. Patients who Exhibit Augmented iNOS and eNOS Report Reduced Survival to Pancreatic Cancer

**Figure 4:**
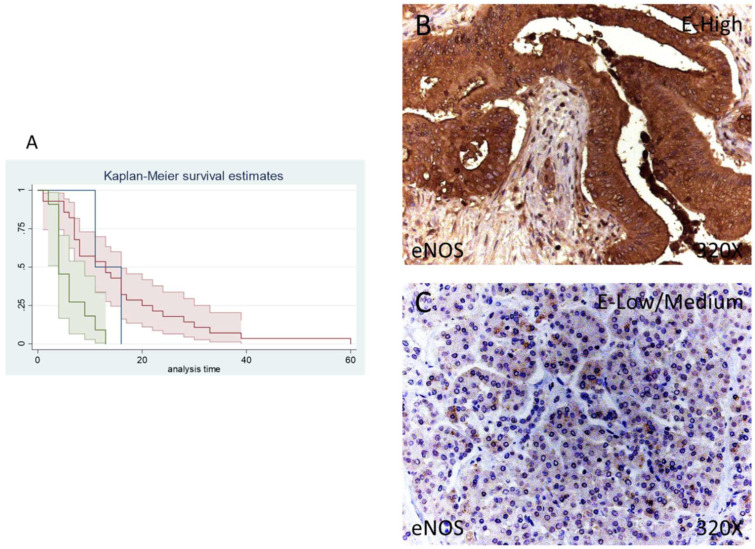
(**A**) Kaplan–Meier curves for survival time according to tumor expression of eNOS. Blue curve = tissue expression classified as negative, red curve = tissue expression classified as low/medium, green curve = tissue expression classified as high. (**B**,**C**) Images showing the protein expression of eNOS in patients diagnosed with pancreatic cancer.

In our immunohistochemical studies, eNOS was expressed in 95.12% of patients whereas 4.88% of the total did not show eNOS expression. A total of 68.29% of patients exhibited a low/moderate eNOS expression while 26.83% of patients presented a high eNOS expression (Figure 4B,C).

The median survival of patients negative for eNOS expression was 11 (11–60) months. Patients with low/moderate eNOS expression showed a median survival of 13 (7–16) months and patients with a high eNOS expression displayed a median survival of 4 (4–9] months. According to our statistical associations, a high expression of eNOS is correlated with a hazard ratio of 2.592 of mortality in comparison to low/moderate expression of this component. The global comparisons reported how the significance value was *p* = 0.001.

**Figure 5:**
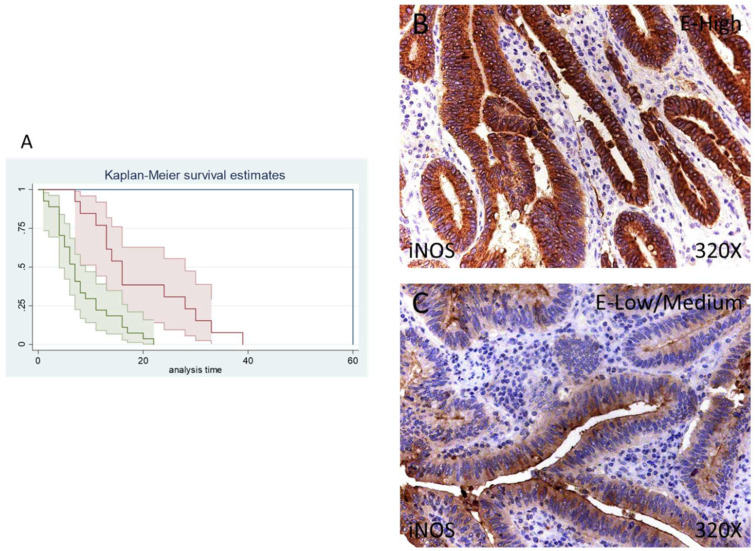
(**A**) Kaplan–Meier curves for survival time according to tumor expression of iNOS. Blue curve = tissue expression classified as negative, red curve = tissue expression classified as low/medium, green curve = tissue expression classified as high. (**B**,**C**) Images showing the protein expression of iNOS in patients diagnosed with pancreatic cancer.

Finally, only 1 patient (2.44%) was negative for iNOS expression, whereas 13 (31.71%) were low/moderate for iNOS and 27 (65.85%) presented a high iNOS expression, according to our immunohistochemical data. (Figure 5B,C).

The median survival of patients with a low/moderate expression of iNOS was 16 (11–28) months, whereas high iNOS was 7 (4–9) months. There was a hazard ratio of 2.337 of mortality in patients with a high iNOS expression. The global comparisons demonstrated how the significance value was *p* < 0.001

## 4. Discussion

The use of histopathological biomarkers has brought potential benefits in the clinical management of pancreatic cancer patients, aiding to provide an accurate prognosis and opening new therapeutic windows [30,31,32,33]. In this study, we have demonstrated a noteworthy elevation of PARP-1, NOX1, NOX2, eNOS and iNOS expression with an important correlation with a low survival rate.

Oxidative stress has been identified as a pivotal agent involved in the pathogenesis of pancreatic cancer, having been proposed as a promising therapeutic target for these patients [34,35]. It seems that enhanced oxidative stress appears to promote an aggressive tumor phenotype, boosting proliferation, apoptosis, and invasion through the regulation of several molecular pathways [20].

In this sense, the augmented detection of PARP-1, NOX1 observed in patients with pancreatic cancer who exhibited a lower survival in our study could be attributed to this fact. PARP-1 is a central molecule closely linked to oxidative stress. On the one hand, PARP-1 expression is necessary to repair oxidative-stress-induced DNA lesions. An augmented expression of PARP-1 can be employed by tumoral cells to protect against the damage produced by oxidative stress [36]. In addition, an upregulated expression of PARP has also been shown to enhance tumor inflammation via the upregulation of NFκB signaling [37]. Because of that, previous studies have demonstrated a direct association between PARP-1 expression in different types of gastrointestinal tumors [38,39]. In this line, PARP-1 inhibitors are being studied in clinics in order to target cancerous cells promoting a deficiency in homologous recombination repair and then cause so-called synthetic lethality [40]. In the case of pancreatic cancer, previous studies have reported that the PARP-1 expression can be used as a marker of differential diagnosis between pancreatic cancer and other pancreatic disorders [41]. Xu et al. [42] found that an elevated cytoplasmic PARP-1 expression conferred therapy resistance and aggressiveness to pancreatic tumoral cells by preventing apoptotic pathways. However, the role of PARP-1 as a prognostic marker of pancreatic cancer is still controversial. For instance, and contrary to other tumors, high nuclear expression of PARP-1 has been associated with longer survival in patients with pancreatic cancer [43] whereas other authors have limited the presence of PARP-1 in acinar normal or tumoral cells but not in normal or cancer ductal cells with no significant survival differences in transgenic mice with pancreatic cancer [44,45]. In this work, we observed that patients with a high expression of PARP-1 are associated with a lower survival. Further studies are needed to validate this result and to unravel the possible use of PARP-1 as a prognostic marker. These things considered, these patients may benefit from the use of different PARP-1 inhibitors in order to limit tumor progression and therapy resistance.

NOX1 and NOX2 are two critical markers also related to oxidative stress. Both enzymes are electron-transporting membrane proteins responsible for ROS generation, mainly superoxide anion (O_2_^●−^), and hydrogen peroxide (H_2_O_2_) [46]. Compelling evidence suggest that NOX1 and other NOX members are prominent players in the carcinogenesis and malignization of tumoral cells, with important actions for their survival and growth [22]. In addition, both NOX1 and NOX2 have important immunomodulatory functions, also being implicated in metastasis. Thus, there are some novel antitumoral therapies targeting these components, showing some promising but still early results [47,48,49]. In this study, we demonstrated that patients with a higher expression of NOX1 and NOX2 exhibit lower survival rates than those with mild or no expression. Recently, Lyu et al. [50] reported an augmented expression of NOX1 and NOX2 in pancreatic cancer tissues, although they failed to find any association between their expression and the patients’ survival. Further efforts are needed to clarify a possible association between NOX1 and NOX2 as prognostic biomarkers in pancreatic tumors.

Finally, we also found a significant association between high levels of eNOS and iNOS expression and a reduced survival of patients with pancreatic cancer. eNOS and iNOS are major sources of reactive nitrogen species (RNS), and eNOS appears to be a critical molecule involved in the carcinogenesis processes and tumor progression, specially via regulation of the PI3K-AKT-eNOS-Ras pathway [51]. Furthermore, compelling evidence has shown that eNOS is upregulated in multiple tumoral lines including pancreatic cancerous cells, with a positive trend towards an increase in the lifespan for mice with genetic eNOS ablation [52]. On the other hand, iNOS is upregulated in a wide variety of tumors, being frequently used as a marker of poor prognosis [53]. In addition, iNOS seems to mediate the therapeutic response of pancreatic tumors to radiotherapy [54]. Despite prior studies not having found any correlation between iNOS and survival, it seems that the higher expression of this component was associated with increased apoptosis, being also related to higher cyclooxygenase 2 (COX-2) expression [55]. In this sense, we have recently observed that patients with high COX-2 expression display a lower survival in comparison to those with mild or no expression [31]. Interestingly, there are some polymorphic variants in eNOS and iNOS genes which represent a potential risk factor for gastric cancer [56]. In pancreatic cancer, it has also been reported that polymorphic variants of eNOS are involved in the development of these tumors [57,58]. Future studies should be conducted to investigate a possible implication of iNOS variants in the risk of suffering from pancreatic cancer and the use of both eNOS and iNOS as therapeutic targets in patients with high expressions of these components.

## 5. Conclusions

The present analysis demonstrated a correlation of a significant increase in the expression of oxidative stress markers (PARP-1, NOX1, NOX2, eNOS and iNOS) with low survival rates, showing high statistical power for both. The 60-month longitudinal surveillance program and the derivative Kaplan–Meier curves indicated low cumulative survival rates. Despite this, further studies are needed to confirm our results in a higher population sample in all situations and compared with acute pancreatitis. Our study may serve to emphasize the value of these molecules as survival-associated biomarkers in pancreatic cancer, giving insight into hopeful therapeutic targets and hence, offering new gates for clinical management.

## Figures and Tables

**Table 1:** Primary antibodies used, together with the dilutions and specifications of the protocol.

Antigen	Species	Clone	Dilution	Provider	Protocol Specifications
NOX1	Rabbit	Polyclonal	1:200	Abcam (ab78016)	10 mM sodium citrate pH = 6 before incubation with blocking solution
NOX2	Goat	Polyclonal	1:400	Abcam (ab111175)	100% Triton 0.1% in PBS, 10 min, before incubation with blocking solution
iNOS	Rabbit	Polyclonal	1:400	Abcam (ab95866)	10 mM sodium citrate pH = 6 before incubation with blocking solution
eNOS	Rabbit	Polyclonal	1:100	Abcam (ab66127)	EDTA pH = 9 before incubation with blocking solution
PARP	Mouse	Monoclonal	1:100	Abcam (ab110915)	10 mM sodium citrate pH = 6 before incubation with blocking solution

## Data Availability

The datasets used and/or analyzed during the present study are available in the manuscript.

## References

[1] Mizrahi J.D., Surana R., Valle J.W., Shroff R.T. (2020). Pancreatic cancer. Lancet.

[2] Rawla P., Sunkara T., Gaduputi V. (2019). Epidemiology of Pancreatic Cancer: Global Trends, Etiology and Risk Factors. World J. Oncol..

[3] (2020). Globocan 2020; International Agency for Research on Cancer; World Health Organization Pancreas. https://gco.iarc.fr/.

[4] Park W., Chawla A., O’Reilly E.M. (2021). Pancreatic Cancer: A Review. JAMA.

[5] Hu J.X., Lin Y.Y., Zhao C.F., Chen W.B., Liu Q.C., Li Q.W., Gao F. (2021). Pancreatic cancer: A review of epidemiology, trend, and risk factors. World J. Gastroenterol..

[6] Zhao Z.Y., Liu W. (2020). Pancreatic Cancer: A Review of Risk Factors, Diagnosis, and Treatment. Technol. Cancer Res. Treat..

[7] Zanini S., Renzi S., Limongi A.R., Bellavite P., Giovinazzo F., Bermano G. (2021). A review of lifestyle and environment risk factors for pancreatic cancer. Eur. J. Cancer.

[8] Vincent A., Herman J., Schulick R., Hruban R.H., Goggins M. (2011). Pancreatic cancer. Lancet.

[9] Kanji Z.S., Gallinger S. (2013). Diagnosis and management of pancreatic cancer. CMAJ.

[10] Takagi K., Imura J., Shimomura A., Noguchi A., Minamisaka T., Tanaka S., Nishida T., Hatta H., Nakajima T. (2020). Establishment of highly invasive pancreatic cancer cell lines and the expression of IL-32. Oncol. Lett..

[11] Haeberle L., Esposito I. (2019). Pathology of pancreatic cancer. Transl. Gastroenterol. Hepatol..

[12] Kurihara K., Hanada K., Shimizu A. (2020). Endoscopic Ultrasonography Diagnosis of Early Pancreatic Cancer. Diagnostics.

[13] Brunner M., Wu Z., Krautz C., Pilarsky C., Grützmann R., Weber G.F. (2019). Current Clinical Strategies of Pancreatic Cancer Treatment and Open Molecular Questions. Int. J. Mol. Sci..

[14] Tonini V., Zanni M. (2021). Pancreatic cancer in 2021: What you need to know to win. World J. Gastroenterol..

[15] Chen X., Liu F., Xue Q., Weng X., Xu F. (2021). Metastatic pancreatic cancer: Mechanisms and detection. Oncol. Rep..

[16] McGuigan A., Kelly P., Turkington R.C., Jones C., Coleman H.G., McCain R.S. (2018). Pancreatic cancer: A review of clinical diagnosis, epidemiology, treatment and outcomes. World J. Gastroenterol..

[17] Yokohira M., Oshima M., Yamakawa K., Ye J., Nakano-Narusawa Y., Haba R., Fukumura Y., Hirabayashi K., Yamaguchi H., Kojima M. (2021). Adequate tissue sampling for the assessment of pathological tumor regression in pancreatic cancer. Sci. Rep..

[18] Reid M.D., Bagci P., Adsay N.V. (2013). Histopathologic assessment of pancreatic cancer: Does one size fit all?. J. Surg. Oncol..

[19] Garcia P.L., Council L.N., Christein J.D., Arnoletti J.P., Heslin M.J., Gamblin T.L., Richardson J.H., Bjornsti M.A., Yoon K.J. (2013). Development and Histopathological Characterization of Tumorgraft Models of Pancreatic Ductal Adenocarcinoma. PLoS ONE.

[20] Martinez-Useros J., Li W., Cabeza-Morales M., Garcia-Foncillas J. (2017). Oxidative Stress: A New Target for Pancreatic Cancer Prognosis and Treatment. J. Clin. Med..

[21] Viera T., Patidar P.L. (2020). DNA damage induced by KP372-1 hyperactivates PARP1 and enhances lethality of pancreatic cancer cells with PARP inhibition. Sci. Rep..

[22] Kamata T. (2009). Roles of Nox1 and other Nox isoforms in cancer development. Cancer Sci..

[23] Garcia V., Sessa W.C. (2019). Endothelial NOS: Perspective and recent developments. Br. J. Pharmacol..

[24] Yang G.Y., Taboada S., Liao J. (2009). Induced nitric oxide synthase as a major player in the oncogenic transformation of inflamed tissue. Methods Mol. Biol..

[25] Ying L., Hofseth L.J. (2007). An emerging role for endothelial nitric oxide synthase in chronic inflammation and cancer. Cancer Res..

[26] Esposito I., Konukiewitz B., Schlitter A.M., Klöppel G. (2014). Pathology of pancreatic ductal adenocarcinoma: Facts, challenges and future developments. World J. Gastroenterol..

[27] Ortega M.A., Sáez M.A., Fraile-Martínez O., Álvarez-Mon M.A., García-Montero C., Guijarro L.G., Asúnsolo Á., Álvarez-Mon M., Bujan J., García-Honduvilla N. (2022). Overexpression of glycolysis markers in placental tissue of pregnant women with chronic venous disease: A histological study. Int. J. Med. Sci..

[28] Ortega M.A., Fraile-Martínez O., García-Montero C., Ruiz-Grande F., Barrena S., Montoya H., Pekarek L., Zoullas S., Alvarez-Mon M.A., Sainz F. (2021). Chronic venous disease patients show increased IRS-4 expression in the great saphenous vein wall. J. Int. Med. Res..

[29] Ortega M.A., Fraile-Martínez O., García-Montero C., Pekarek L., Alvarez-Mon M.A., Guijarro L.G., del Carmen Boyano M., Sainz F., Álvarez-Mon M., Buján J. (2021). Tissue remodelling and increased DNA damage in patients with incompetent valves in chronic venous insufficiency. J. Cell. Mol. Med..

[30] Dell’Aquila E., Fulgenzi C.A.M., Minelli A., Citarella F., Stellato M., Pantano F., Russano M., Cursano M.C., Napolitano A., Zeppola T. (2020). Prognostic and predictive factors in pancreatic cancer. Oncotarget.

[31] Ortega M.A., Pekarek L., Garcia-Montero C., Fraile-Martinez O., Saez M.A., Asúnsolo A., Alvarez-Mon M.A., Monserrat J., Coca S., Toledo-Lobo M.V. (2022). Prognostic role of IRS-4 in the survival of patients with pancreatic cancer. Histol. Histopathol..

[32] Cardiff R.D., Gregg J.P., Miller J.W., Axelrod D.E., Borowsky A.D. (2006). Histopathology as a Predictive Biomarker: Strengths and Limitations. J. Nutr..

[33] Pekarek L., Fraile-Martinez O., Garcia-Montero C., Alvarez-Mon M.A., Acero J., Ruiz-Llorente L., García-Honduvilla N., Albillos A., Buján J., Alvarez-Mon M. (2021). Towards an updated view on the clinical management of pancreatic adenocarcinoma: Current and future perspectives. Oncol. Lett..

[34] Costello E., Greenhalf W., Neoptolemos J.P. (2012). New biomarkers and targets in pancreatic cancer and their application to treatment. Nat. Rev. Gastroenterol. Hepatol..

[35] Yu J.H., Kim H. (2014). Oxidative stress and cytokines in the pathogenesis of pancreatic cancer. J. Cancer Prev..

[36] Hou D., Liu Z., Xu X., Liu Q., Zhang X., Kong B., Wei J.J., Gong Y., Shao C. (2018). Increased oxidative stress mediates the antitumor effect of PARP inhibition in ovarian cancer. Redox Biol..

[37] Swindall A.F., Stanley J.A., Yang E.S. (2013). PARP-1: Friend or Foe of DNA Damage and Repair in Tumorigenesis?. Cancers.

[38] Liu Y., Zhang Y., Zhao Y., Gao D., Xing J., Liu H. (2016). High PARP-1 expression is associated with tumor invasion and poor prognosis in gastric cancer. Oncol. Lett..

[39] Alhadheq A.M., Purusottapatnam Shaik J., Alamri A., Aljebreen A.M., Alharbi O., Almadi M.A., Alhadeq F., Azzam N.A., Semlali A., Alanazi M. (2016). The Effect of Poly(ADP-ribose) Polymerase-1 Gene 3′Untranslated Region Polymorphism in Colorectal Cancer Risk among Saudi Cohort. Dis. Markers.

[40] Zhu H., Wei M., Xu J., Hua J., Liang C., Meng Q., Zhang Y., Liu J., Zhang B., Yu X. (2020). PARP inhibitors in pancreatic cancer: Molecular mechanisms and clinical applications. Mol. Cancer.

[41] Castiglione R., Calogero A.E., Vicari E., Calabrini G., Cosentino A., D’Agati P., Fraggetta F., Salemi M. (2020). Poly (ADP-Ribose) Polymerase 1 Protein Expression in Normal Pancreas and Pancreatic Adenocarcinoma. Case Rep. Gastrointest. Med..

[42] Xu F., Sun Y., Yang S.Z., Zhou T., Jhala N., McDonald J., Chen Y. (2019). Cytoplasmic PARP-1 promotes pancreatic cancer tumorigenesis and resistance. Int. J. Cancer.

[43] Klauschen F., von Winterfeld M., Stenzinger A., Sinn B.V., Budczies J., Kamphues C., Bahra M., Wittschieber D., Weichert W., Striefler J. (2012). High nuclear poly-(ADP-ribose)-polymerase expression is prognostic of improved survival in pancreatic cancer. Histopathology.

[44] Martínez-Bosch N., Fernández-Zapico M.E., Navarro P., Yélamos J. (2016). Poly(ADP-Ribose) Polymerases: New Players in the Pathogenesis of Exocrine Pancreatic Diseases. Am. J. Pathol..

[45] Tuli R., Shiao S.L., Nissen N., Tighiouart M., Kim S., Osipov A., Bryant M., Ristow L., Placencio-Hickok V., Hoffman D. (2019). A phase 1 study of veliparib, a PARP-1/2 inhibitor, with gemcitabine and radiotherapy in locally advanced pancreatic cancer. EBioMedicine.

[46] Tarafdar A., Pula G. (2018). The Role of NADPH Oxidases and Oxidative Stress in Neurodegenerative Disorders. Int. J. Mol. Sci..

[47] Stalin J., Garrido-Urbani S., Heitz F., Szyndralewiez C., Jemelin S., Coquoz O., Ruegg C., Imhof B.A. (2019). Inhibition of host NOX1 blocks tumor growth and enhances checkpoint inhibitor-based immunotherapy. Life Sci. Alliance.

[48] Martner A., Aydin E., Hellstrand K. (2019). NOX2 in autoimmunity, tumor growth and metastasis. J. Pathol..

[49] Aydin E., Hallner A., Grauers Wiktorin H., Staffas A., Hellstrand K., Martner A. (2018). NOX2 inhibition reduces oxidative stress and prolongs survival in murine KRAS-induced myeloproliferative disease. Oncogene.

[50] Lyu P.W., Xu X.D., Zong K., Qiu X.G. (2022). Overexpression of DUOX2 mediates doxorubicin resistance and predicts prognosis of pancreatic cancer. Gland Surg..

[51] Lim K.H., Ancrile B.B., Kashatus D.F., Counter C.M. (2008). Tumour maintenance is mediated by eNOS. Nature.

[52] Lampson B.L., Kendall S.D.S., Ancrile B.B., Morrison M.M., Shealy M.J., Barrientos K.S., Crowe M.S., Kashatus D.F., White R.R., Gurley S.B. (2012). Targeting eNOS in Pancreatic Cancer. Cancer Res..

[53] Vanini F., Kashfi K., Nath N. (2015). The dual role of iNOS in cancer. Redox Biol..

[54] Pereira P.M.R., Edwards K.J., Mandleywala K., Carter L.M., Escorcia F.E., Campesato L.F., Cornejo M., Abma L., Mohsen A.A., Iacobuzio-Donahue C.A. (2020). iNOS Regulates the Therapeutic Response of Pancreatic Cancer Cells to Radiotherapy. Cancer Res..

[55] Kong G., Kim E.K., Kim W.S., Lee K.T., Lee Y.W., Lee J.K., Paik S.W., Rhee J.C. (2002). Role of cyclooxygenase-2 and inducible nitric oxide synthase in pancreatic cancer. J. Gastroenterol. Hepatol..

[56] Zhu Y., Jiang H., Chen Z., Lu B., Li J., Peng Y., Shen X. (2018). The genetic association between iNOS and eNOS polymorphisms and gastric cancer risk: A meta-analysis. Onco. Targets Ther..

[57] Dagmura H., Yigit S., Gumusay O., Nursal A.F., Daldal E., Karakus N. (2021). eNOS and VEGF Variants Might Increase the Risk of Pancreatic Cancer. Cytol. Genet..

[58] Ortega M.A., Pekarek L., Fraile-Martinez O., Garcia-Montero C., Saez M.A., Asúnsolo A., Alvarez-Mon M.A., Monserrat J., Ruiz-Llorente L., García-Honduvilla N. (2022). Implication of ERBB2 as a Predictive Tool for Survival in Patients with Pancreatic Cancer in Histological Studies. Curr. Oncol..

